# Potential of the Endophytic Fungus *Phialocephala* *fortinii* Rac56 Found in *Rhodiola* Plants to Produce Salidroside and *p*-Tyrosol

**DOI:** 10.3390/molecules21040502

**Published:** 2016-04-16

**Authors:** Jinlong Cui, Tingting Guo, Jianbin Chao, Mengliang Wang, Junhong Wang

**Affiliations:** Institute of Applied Chemistry, Shanxi University, Taiyuan 030006, China; CJL717@sxu.edu.cn (J.C.); guott880118@163.com (T.G.); chao@sxu.edu.cn (J.C.); wangjunh@sxu.edu.cn (J.W.)

**Keywords:** *Phialocephala fortinii*, fungal bioactivity, *Rhodiola angusta*, accumulation of active component

## Abstract

2-(4-Hydroxyphenyl)ehyl-β-d-glucopyranoside (salidroside) and 4-(2-hydroxyethyl)phenol (*p*-tyrosol) are famous food and medicine additives originally derived from alpine *Rhodiola* plants. Salidroside or *p*-tyrosol production by the endophytic fungus Rac56 (*Phialocephala fortinii*) was confirmed by UPLC/Q-TOF-MS and ^1^H-NMR. The fermentation conditions were optimized by orthogonal design using data processing system software. The broth fermentation results showed that salidroside and *p*-tyrosol extraction yields from Rac56 were stable and reached 1.729 ± 0.06 mg and 1.990 ± 0.05 mg per mL of culture medium, respectively. The optimal conditions for salidroside and *p*-tyrosol production in fermentation culture of Rac56 were determined to be 25 °C, pH values of 7 and 5, Czapek-Dox culture medium volumes of 150 mL and 50 mL in 250 mL flasks, rotation speeds of 100× g and 200× g, and fermentation durations of 7 and 15 days, respectively. Under these optimal conditions, stable yields of 2.339 ± 0.1093 mg and 2.002 ± 0.0009 mg per mL of culture medium of salidroside and *p*-tyrosol, respectively, were obtained, indicating that the *P. fortinii* Rac56 strain is a promising source of these compounds.

## 1. Introduction

**Figure 1:**
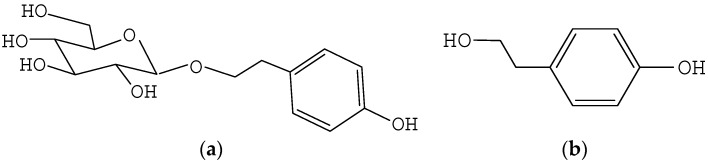
Chemical structures of two active compounds in *Rhodiola* plants. (**a**) Salidroside; (**b**) *p*-tyrosol.

Salidroside (SAL) and *p*-tyrosol (TYR) (Figure 1) are the main bioactive ingredients of all *Rhodiola* spp., such as *R. rosea* and *R. crenulata* [1], which is a rare and valuable plant of the Crassulaceae family found in high-altitude (>2500 m) regions [2]. It was used as food crop and folk medicine by many ancient civilizations because of its pharmacologic effects, such as antioxidant, anti-fatigue, anti-aging, as well as adaptogenic properties [3]. With increasing demand for SAL and TYR as additives in the health food, cosmetic, and pharmaceutical industries, wild *Rhodiola* plants are being over-exploited [4]. However, artificial cultivation of *Rhodiola* plants has been limited by the elevation requirement of planting areas, long production cycle, and low content of active components [4]. In recent years, tissue and cell culture as well as chemical synthesis methods have been used for SAL and TYR production; however, these methods are costly, low yielding, and conducive to pollution [5]. As a result, the prices of SAL and TYR are high and their supplies are limited.

Endophytes are microorganisms that live specifically within the tissue of their host plants without causing apparent disease symptoms [6]. Studies have shown that all investigated plants harbor endophytic fungi [7,8]. During long-term co-evolution, complex interaction and cross-talk between endophytic fungi and their hosts, such as signal exchange and horizontal gene transmission [9,10], have occurred to jointly cope with biotic and abiotic stresses [11,12]. Since a taxol-producing endophytic fungus *Taxomyces* *andreanae* was isolated from its host plant *Taxus* *brevifolia* in 1993 [13], follow-up studies have further confirmed that endophytic fungi and their host plant might have the same synthetic routes of some secondary metabolites and could produce similar or identical metabolites [14]. In recent years, many plant-derived bioactive products have been isolated from endophytic fungi [15], making them more economical and easier to produce at a larger scale by submerged fermentation.

In the course of our continuous search for endophytic fungi in the three main species of *Rhodiola* in China, a total of 347 isolates were obtained for the first time, from which SAL and TYR-producing fungi were screened. Four isolates were found to be potential candidates, but only one isolate Rac56 was determined to be a remarkable producer of SAL and TYR. The current study is the first to report *Rhodiola* endophytic fungus and optimize its fermentation conditions, with results showing that the isolate could serve as a reliable source for SAL and TYR production.

## 2. Results

### 2.1. Isolation and Screening of SAL- and TYR-Producing Fungi

A total of 347 endophytic fungi were obtained from three *Rhodiola* species, and about 180 representative morphotypes (71, 57, and 52 isolates from *R. crenulata*, *R. angusta*, and *R. sachalinensis*, respectively) were fermented to determine the SAL and TYR content. The high performance liquid chromography (HPLC) analyses results showed that four endophytic fungi (Rac12, Rac56, and Rac63 from *R. angusta*; Rct30 from *R. crenulata*) can produce SAL or TYR (Table 1). Figure S1 shows the ultraviolet absorption and HPLC chromatograms of SAL and TYR standards, and the extracts of Rac12 (mycelial extract), Rct30 (mycelial extract), Rac56 and Rac63 (filtrate extract), respectively. Rac56 could produce an average of 1.729 ± 0.063 mg SAL and 1.990 ± 0.050 mg TYR, and each of the other three isolates produced an average of ≤0.402 ± 0.248 mg SAL or TYR per mL of culture medium (Table 1). Furthermore, production of SAL and TYR from most isolates declined even after being sub-cultured several times in the follow-up studies; only Rac56 remained the most stable and had the highest yield, with the yield in *culture* filtrate shown to be higher than that in mycelia. Therefore, the fermentation broth extract of Rac56 was chosen for further studies.

### 2.2. Identification of Rac56 as the Ideal SAL- and TYR-Producing Fungus

**Figure 2:**
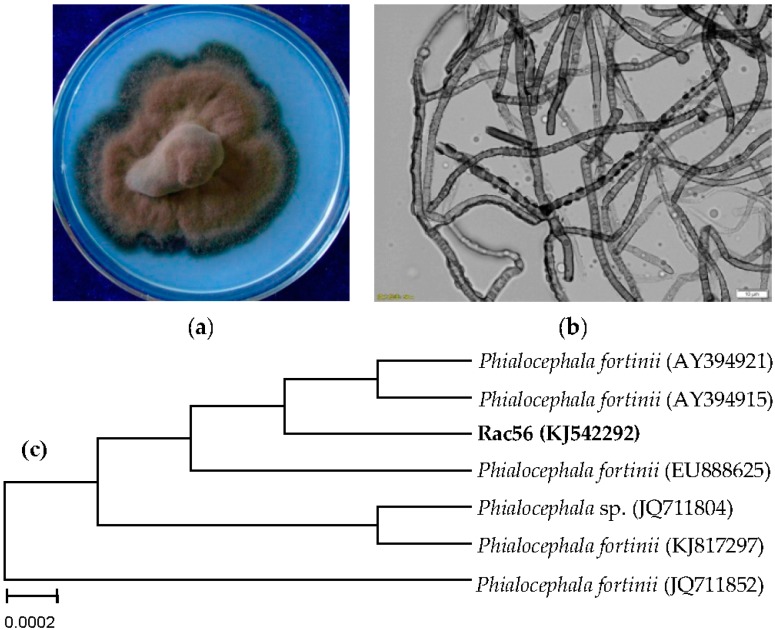
Identification of endophytic fungus Rac56 from *Rhodiola* *angusta*. Morphological analyses of the colony characteristics (**a**) and fungal mycelium (**b**) were conducted 20 days post culturing. Under a microscope, the mycelia were observed to be dark with a size range of 1.5–4.5 µm. Aneighbor-joining tree (**c**) based on ITS rDNA sequence of Rct56 and its closest ITS rDNA matches in GenBank was constructed using MEGA 4.0 software based on 1000 bootstrap replicates.

The colony diameter of Rac56 was 45 mm after inoculation in the PDA medium at 25 ± 2 °C for 20 days, with gray-black color, central uplift, neat edge, no visible secretion in medium, and a black melanized outer edge. The mycelia were dense, appeared floss-shaped, and had no evident substrate mycelium (Figure 2a). Under a microscope, the hypha was dark colored and had transverse septa, which belong to dark septate endophytes (DSE), had a diameter range of 1.5–4.5 µm, abundant globular particle contents in hypha with terminal expansion, and did not produce conidium (Figure 2b). The identification was authenticated by ITS analyses. The rDNA sequence of Rac56 (KJ542292, 519 bp) and its closest related species available at GenBank were selected for construction of NJ phylogenetic tree using MEGA 4.0 program after sequence alignment with Clustal W. The endophytic fungus Rac56 was found to be closely related to *Phialocephala* *fortinii* (AY394915 and AY394921) (Figure 2c) with 99% homology. The morphological characteristic described above corresponded to anamorphic *P. fortinii* [16,17,18,19,20], which led to its designation as *P. fortinii*.

### 2.3. Validation of SAL and TYR Produced from Rac56 by LC-MS and ^1^H-NMR

**Figure 3:**
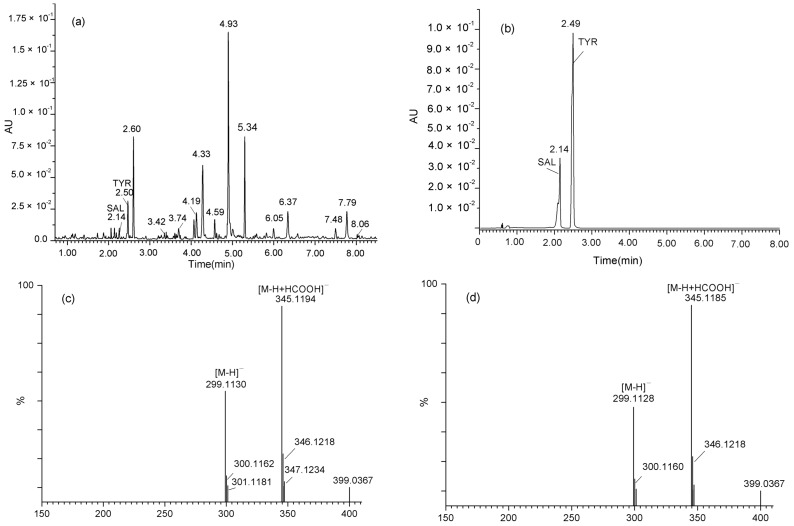
UV profile and MS chromatogram of the culture filtrate the Rac56 strain and commercially available salidroside (SAL) and *p*-tyrosol (TYR). (**a**) UV chromatograms of culture filtrate of fungus Rac56 containing SAL (Rt, 2.15) and TYR (Rt, 2.51) detected at a wavelength of 277 nm; (**b**) UV chromatograms of authentic SAL (Rt, 2.14) and TYR (Rt, 2.49) monitored by UPLC-UV detector at the wavelength of 277 nm; (**c**) Molecular ion peak at *m*/*z* 299.1130 [M − H]^−^ and *m*/*z* 345.1194 [M − H + HCOOH]^−^ of SAL produced by Rac56; (**d**) Molecular ion peak [M − H]^−^ at *m*/*z* 299.1128 and [M − H + HCOOH]^−^ at *m*/*z* 345.1185 of authentic SAL.

SAL and TYR produced by the Rac56 strain were further identified by UPLC/Q-TOF-MS. Results showed that the retention times (Rt) of the two metabolites produced by Rac56 were 2.15 min and 2.51 min (Figure 3a), corresponding to 2.14 min and 2.49 min of standard SAL and TYR in the UV profile (Figure 3b), respectively; the molecular ion at *m*/*z* 299.1130 [M − H]^−^ and *m/z* 345.1194 [M − H + HCOOH]^－^of the chemical component produced by Rac56 (Figure 3c) was consistent with the molecular ion at *m*/*z* 299.1128 [M − H]^−^ and *m*/*z* 345.1185 [M − H + HCOOH]^−^ of standard SAL (Figure 3d).

**Figure 4:**
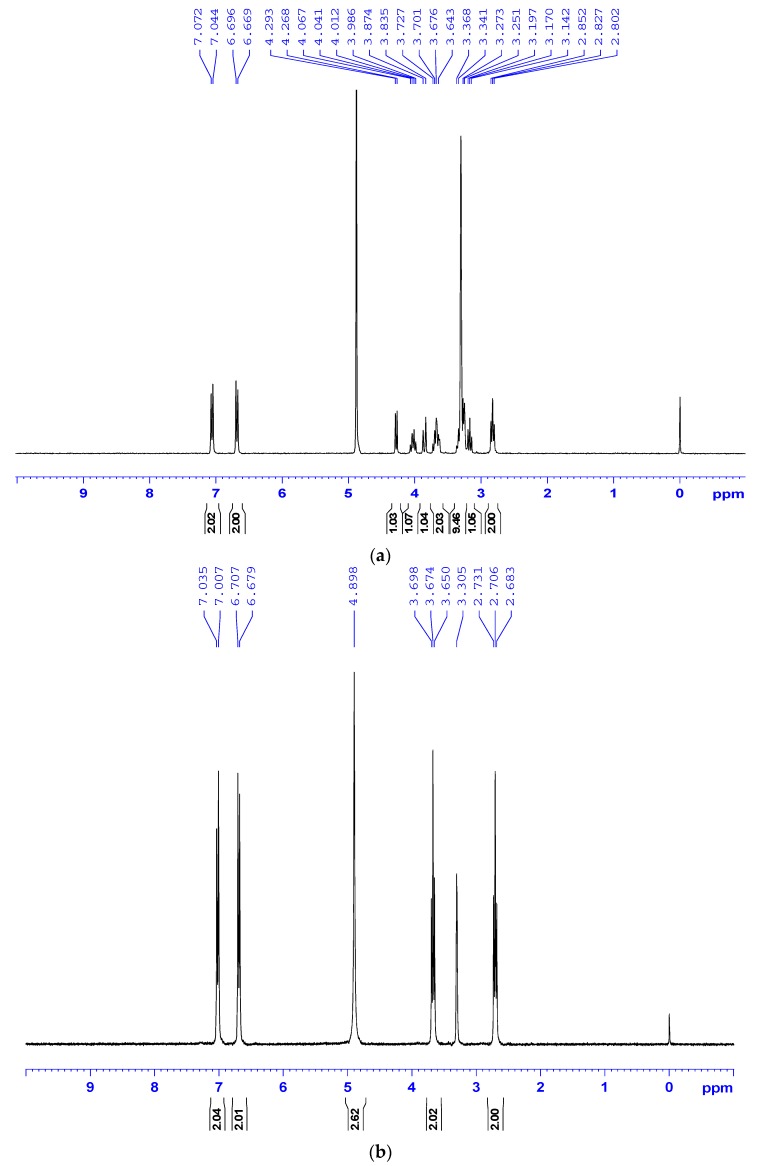

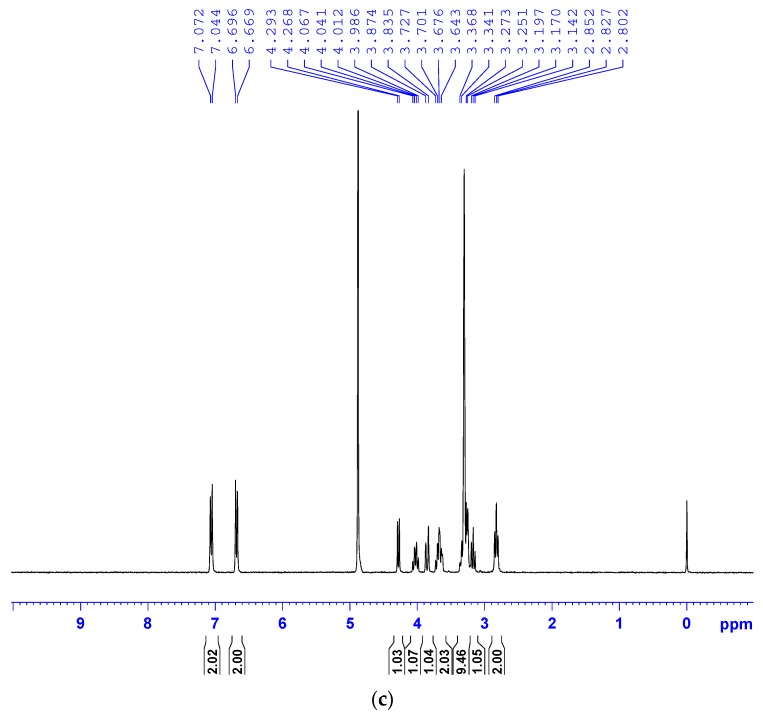
^1^H-NMR spectra of samples. ^1^H-NMR spectra of standard/commercially available SAL (**a**) and TYR (**b**); and the chemicals in culture filtrate extract from Rac56 (**c**). All test samples were normalized to solvent CD_3_OD signal, in accordance with [4,21].

However, molecular ion peaks of TYR could not be obtained from the MS spectrum by using either negative-ion or positive ion mode although it could be detected by the UV profile of both the TYR standard and the Rac56 sample. The fungal SAL and TYR produced by the Rac56 strain were examined by ^1^H-NMR spectroscopic analysis. The aromatic region (δ_H_ 6.5–7.2 ppm, H3/5 and H2/6 proton signals for SAL and TYR were 6.982 ppm to 7.009 ppm) and the sugar region (δ_H_ 3–4.5 ppm, H^−1^ proton signal for SAL was 4.218 ppm to 4.248 ppm) were found to contain aromatic compound and glycosidic constituent peaks identical to those found in commercially available SAL and TYR, respectively (Figure 4).These findings are also consistent with previous studies [4,21] and lend further support to the authenticity of SAL and TYR produced by the Rac56 strain.

### 2.4. Fermentation and Optimal Growth Conditioning for SAL and TYR Production from Rac56

Results of HPLC quantification of SAL and TYR indicated that the yields of SAL cultured with Czapek-Dox medium (CD), potato dextrose medium (PD), Sabouraud medium (SD), and lactose-beef extract-peptone medium (LBP) media were 1.715 ± 0.007 mg/mL, 1.368 ± 0.021 mg/mL, 1.046 ± 0.018 mg/mL and 1.140 ± 0.023 mg/mL, respectively, and the yields of TYR in CD (1.863 ± 0.041 mg/mL) was higher than that in PD (1.551 ± 0.030 mg/mL), SD (1.419 ± 0.008 mg/mL), or LBP (1.067 ± 0.016 mg/mL). Thus, the CD medium was chosen as the best medium for subsequent optimization of the fermentation conditions.

Table 2, Table 3 and Table 4 present the SAL and TYR production optimization using a five factor and four levels (L16(4^5^)) orthogonal experimental design. The results of *F* test showed that temperature, culture volume, and fermentation time had significant effects on SAL and TYR production. The significance of each factor was determined by the corresponding *F* values and *P* values (Table 2 and Table 3). *p* values of *p* ≤ 0.05 and *p* ≤ 0.01 were considered statistically significant and highly significant, respectively (Table 2 and Table 3). Based on the highest values of SAL production, the optimal fermentation conditions were 25 °C, initial pH 7.0, 150 mL for culture volume in a 250 mL flask, 100× g of rotation speed, and seven days of fermentation time. The optimal condition for TYR production was set as; temperature of 25 °C, initial pH of 5.0, culture volume of 50 mL in a 250 mL flask, rotation speed of 200× *g*, and fermentation time of 15 days. With the optimal conditions outlined above, SAL and TYR yields of 2.3392 ± 0.1093 mg and 2.0018 ± 0.0009 mg per mL of culture medium, respectively, were obtained. These yields were higher than those of any other conditions (Table 4).

## 3. Discussion

Endophytic fungi are increasingly being recognized for their huge potential in the production of biologically active compounds for food, agriculture, and medicine [22]. In the present study, four endophytic fungi, namely, Rac12, Rct30, Rac56, and Rac63, were found to produce SAL or TYR. They were identified as *Lachnum* sp., *Neonectriaramulariae* and *P. fortinii*, respectively. Previously these endophytic fungi were also isolated from other plants and found to possess versatile capacities of producing biologically active compounds [17,23,24]. However, this study is the first to report the presence of these fungi in *Rhodiola* spp. and to reveal the existence of fungal endophytes with SAL- or TYR-producing abilities in nature. Taken together, our findings support the co-evolution theory of symbiosis between endophytic fungi and their hosts [9]. Furthermore, our findings provide a promising method of SAL and TYR production that is easy, sustainable, and low-cost, which are important factors not only in meeting the demands of SAL and TYR production using *Rhodiola* plants but also in protecting these medicinally important plant species. 

Among the four fungi we studied, the Rac56 isolate exhibited the most stable and highest yield of SAL and TYR. By ITS sequence based genotyping we found our isolate Rac56 to be similar to *P. fortinii*, (Ascomycota, pezizomycotina, helotiales) a typical DSE fungus which is a widespread fungal endophyte of plants [16,18]. Several reports have shown that *P. fortinii* exhibits versatile capacities of promoting elemental nitrogen (N) and phosphorus (P) absorption by host plants [25] and synthesizing glucose [26] by co-existing with host roots. Further, *P. fortinii* have also been shown to aid the enhancement of disease resistance in hosts, controlling *Verticillium yellows* in Chinese cabbage [27], the capacity to produce chemicals such as hydroxamate siderophore [28] and other enzymes in improving host resistance and absorption of nutrients, for example, production of extracellular aryl sulfatase to decompose aryl sulphates [29]. All of these abilities, along with its ability to produce SAL and TYR, suggest that *P. fortinii* plays a key role in symbiosis with host plants.

SAL and TYR were originally derived from *Rhodiola* plants, and were synthesized from phenylalanine or tyrosine in the cinnamic acid pathway (originally called the shikimate pathway) [30]. TYR is synthesized first and serves as a precursor for SAL; in fact, SAL is the 8-*O*-β-d-glucoside of TYR and its glycosylation is dependent on UDP-glucosyltransferase catalysis [5,31]. As closely related secondary metabolites of the cinnamic acid pathway, SAL and TYR are always detected together, and their yields were found to be stable in *Rhodiola* plants [32]. However, it is not yet known whether the same pathway is involved in SAL and TYR biosynthesis in fungi and their hosts, or whether the genes involved in SAL and TYR biosynthesis were horizontally transferred from the host and probably mutated during the course of evolution, which may explain the stabilized or unstabilized yields of SAL and TYR after repeated sub-culturing in certain endophytic strains. Such questions are of significant interest for further elucidating the interaction between endophytic fungi and their hosts, and regulating SAL and TYR production efficiency and metabolic optimization.

## 4. Materials and Methods

### 4.1. Plant Materials and Isolation of Endophytic Fungi

*R. crenulata* samples were collected from Mila Pass (altitude 5007 m, E 92°22′31″–92°24′88″, N 29°44′33″–29°46′56″), and *R. sachalinensis* and *R. angusta* were collected from Changbai Mountain (altitude 2200 m, E 128°08′17″–128°11′10″, N 44°11′20″–44°13′29″) of China, and they were identified by Jinlong Cui and voucher specimens were deposited in the Institute of Applied Chemistry, Shanxi University. Isolation and purification of fungus from plant samples was done as described earlier in Sun *et al.* [12]. 

### 4.2. Fermentation and Secondary Metabolite Extraction Fromendophytic Fungal spp.

Six pieces (5 mm diameter) of mycelial agar plugs from the edge of growing cultures of endophytic fungi were each inoculated into 500 mL Erlenmeyer flask with 200 mL of liquid CD medium (NaNO_3_, 3 g; K_2_HPO_3_, 1 g; MgSO_4_·7H_2_O, 0.5 g; KCl, 0.5 g; FeSO_4_, 0.01 g; sucrose, 30 g; and deionized water to a total volume of 1000 mL). All flasks were incubated under constant shaking (150× *g*) at 25 ± 2 °C for 10 days. The fungal broth was filtered to separate the culture broth and mycelia using Whatman No. 1 filter paper. The mycelia and filtrate were extracted following the SAL and TYR extraction method as described in Chinese Pharmacopoeia [33]. The mycelia were dried, powdered, weighed, and extracted three times with 10-fold (*w*/*v*) methanol by ultrasonication for 30 min, after which the three methanol extracts were combined. The culture filtrate was then evaporated under reduced pressure (8 × 10^3^ Pa) to yield an extract, which was weighed and extracted three times with 10-fold (*w*/*v*) methanol for 10 min and extracts pooled. The extraction solution was then filtered using Whatman No. 1 filter paper and centrifuged at 5000× *g* for 10 min. Lastly, the supernatants were harvested to obtain the mycelia and filtrate extracts.

### 4.3. Screening of SAL and TYR-Producing Endophytic Fungi

The mycelia and filtrate extracts prepared were filtered through a 0.45 µm Millex-HV filter membrane (Millipore, Billerica, MA, USA) before determination of SAL and TYR contents by RP-HPLC analyses based on the method described in Chinese Pharmacopoeia [33]. The analytical HPLC system (Agilent 1200 LC Series, Santa Clara, CA, USA) was equipped with a quaternary pump with vacuum degasser, an Eclipse XDB-C_18_ (4.6 × 250 mm, 5 µm, Agilent), a 10 µL manual injector, a thermostatted column compartment, and a UV detector. The mobile phase consisted of a methanol/water mixture (30:70) at a flow rate of 1.0 mL/min at 30 °C and monitored using a UV detector at 276 nm. HPLC-grade methanol was purchased from Fisher Scientific (Pittsburgh, PA, USA). Ultrapure water was prepared using an ultrapure water system from Arium Comfort I (Sartorius, Göttingen, Germany). The SAL and TYR standards were purchased from the National Institute for the Control of Pharmaceutical and Biological Products (Beijing, China). Detections of SAL and TYR in fungal extracts were based on their retention times. Quantifications of SAL and TYR were done using a linear regression equation fitted in a calibration graph as y = 0.88325x + 0.44806 (r = 0.99951) and y = 2.59700x + 2.18000 (r = 0.99960), respectively. The concentration ranges of SAL and TYR were 2 µg/mL to 200 µg/mL and 1.6 µg/mL to 160 µg/mL, respectively.

### 4.4. Molecular Weight Determination by UPLC/Q-TOF-MS

The identity of SAL and TYR produced by the fungi was confirmed by determination of their molecular masses using ultra-performance liquid chromatography/quadrupole time–of–flight mass spectrometry (UPLC/Q-TOF-MS) analyses. Analytical UPLC separation was performed on a Waters Acquity I-Class system (Waters Corp., Milford, MA, USA), equipped with a UV detector, a BEH C_18_ column (2.1 × 100 mm, 1.7 µm), a quaternary pump, and an autosampler. The mobile phase contained two solutions, namely (A) 0.1% (*v*/*v*) formic acid in high-purity water and (B) chromatographic grade acetonitrile. A timed gradient program of T (min)/B (%) (0/5, 9/35, 12/70, 12.5/5, 15/5) was used for elution, with a flow rate of 0.4 mL/min. The column temperature was maintained at 40 °C, the sample injection volume was 3 µL, and the detection wavelength was set to 277 nm. MS analyses were performed on a Waters Xevo G2 Q-TOF (Micromass MS Technologies, Manchester, UK), equipped with an electrospray ionization source. Lock Spray™ was used to ensure mass accuracy and reproducibility. The deprotonated leucine-enkephalin (C_28_H_37_N_5_O_7_) at *m/z* 554.2615 was used as the lock mass in negative-ion detection mode. The source block and desolvation lamp were kept at 100 °C and 400 °C, respectively. The nebulization gas was set to 800 L/h at 400 °C. The cone gas was 20 L/h, and the source temperature was 100 °C. The capillary voltage and cone voltage were set at 2700 V and 40 V, respectively. The collision energy was 6 V, and the MS data were acquired from a mass-to-charge ratio (*m/z*) range of 100–1500. MS/MS experiments were performed by ramping collision energies from 35 V to 45 V. A dwelling time of 0.2 s was employed with an inter-acquisition delay of 0.01 s. Data acquisition and processing were performed using Masslynx v 4.1 software (Version 9.50; Science Press, Beijing, China).

### 4.5. Structure Determination of SAL and TYR in Fungal Extracts by ^1^H-NMR 

To confirm the authenticity of fungal SAL and TYR, ^1^H nuclear magnetic resonance (^1^H-NMR) spectra were determined on dried fungal extracts dissolved in methanol-*d*_4_ (CD_3_OD) using an AVANCE 300 MHz spectrometer (Bruker, Fallanden, Switzerland). Chemical shifts were reported as δ values relative to tetramethylsilane used as an internal reference. Spectra related compounds were assigned by comparing fungal extracts and authentic SAL and TYR in CD_3_OD following the method in reference [4,21].

### 4.6. Identification of SAL and TYR-Producing Fungus 

Morphological identification of SAL and TYR-producing fungi was based on colony appearance and sporulation. Mycelial characteristics, such as color, septum, transparency, shape, and sporogenous structure and spores’ characteristics, such as shape, size, and conidial fructification, were observed through specimens mounting in 2% KOH under a light microscope (BX53, Olympus, Tokyo, Japan). To authenticate the results of morphological identification, molecular method was adopted to verify the taxonomic status of fungi. The ITS sequence (ITS1: TCCGTAGGTGAACCTGCGG; ITS4: TCCTCCGCTTATTGATATGC) was subjected to sequence similarity search performed on the NCBI database (http://www.ncbi.nlm.gov/BLAST). Sequences with high homology with the submitted isolate were retrieved and analyzed using Clustal W, and a neighbor-joining (NJ) phylogenetic tree was constructed from the evolutionary distance data using MEGA 4.0. The ITS rDNA sequences of the Rac12, Rac30, Rac56, and Rac63 strains that were identified by the molecular method are deposited in GenBank under accession numbers KJ542268, KJ542219, KJ542292, and KJ542358, respectively. 

### 4.7. Optimization of Fermentation Conditions for SAL and TYR Production from Rac56 

Single-factor design was employed to evaluate the effects of four culture media: CD, PD (potato, 200 g; glucose, 20 g; deionized water, 1000 mL), SD (peptone, 10 g; glucose, 40 g; deionized water, 1000 mL), and LBP(lactose, 5 g; beef extract, 5 g; yeast extract, 5 g; peptone, 10 g; glucose, 10 g; NaCl, 5 g; deionized water, 1000 mL; pH 6.8 ± 0.2) on SAL and TYR production by Rac56. An orthogonal design of five factors and four levels (L16(4^5^)) was adopted to optimize the culture conditions for SAL and TYR production using Data Processing System software (Version 9.50; Science Press, Beijing, China), as shown in Table 5. The fermentation broths were extracted, and SAL and TYR yields were determined to obtain the optimal fermentation parameters for an increased SAL and TYR yield based on the methods outlined in this study.

### 4.8. Statistical Analysis

All experiments were performed in triplicate and data are presented as mean ± standard deviation (SD). Statistical difference was analyzed with multi-way ANOVA using DPS 9.50 software (Science Press, Beijing, China).

## Figures and Tables

**Table 1:** SAL and TYR production from endophytic fungi (*Rhodiola* plants).

Name	Rac12 (mg/mL)		Rct30 (mg/mL)		Rac56 (mg/mL)		Rac63 (mg/mL)	
	Fil. Ext. ^3^	Myc. Ext. ^4^	Fil. Ext.	Myc. Ext.	Fil. Ext.	Myc. Ext.	Fil. Ext.	Myc. Ext.
SAL ^1^	－	0.131 ± 0.009	－	0.343 ± 0.465	1.729 ± 0.063	0.028 ± 0.032	0.402 ± 0.248	－
TYR ^2^	－	0.113 ± 0.010	－	－	1.990 ± 0.050	－	0.083 ± 0.117	－

^1^ Salidroside; ^2^ *p*-tyrosol; ^3^ Filtrate extract; ^4^ Mycelial extract.

**Table 2:** Test of significant effects of five factors on fermentation for SAL production from Rac56 using Data Processing System.

^1^ SAL											
T_1_	19.298	19.086	19.471	19.1383	18.4791	^2^ V	SS	df	S^2^	*F*	*p*
T_2_	24.2256	19.8705	19.1425	18.6516	21.8906	^3^ TE	3.6368	3	1.2123	40.3207	0.0001
T_3_	15.607	18.4464	17.0162	18.4303	17.2551	^4^ pH	0.106	3	0.0353	1.1751	0.3346
T_4_	16.8242	18.5519	20.3251	19.7346	18.33	^5^ MV	0.4944	3	0.1648	5.4815	0.0037
*x*_1_	1.6082	1.5905	1.6226	1.5949	1.5399	^6^ RS	0.0837	3	0.0279	0.9278	0.4386
*x*_2_	2.0188	1.6559	1.5952	1.5543	1.8242	^7^ FT	1.01	3	0.3367	11.1977	0.0001
*x*_3_	1.3006	1.5372	1.418	1.5359	1.4379	error	0.9621	32	0.0301		
*x*_4_	1.402	1.546	1.6938	1.6446	1.5275	*F*_0.05_	2.90				
R	0.7182	0.1187	0.2757	0.1087	0.3863	*F*_0.01_	4.46				

^1^ Salidroside, ^2^ Source of Variation, ^3^ Temperature, ^4^ Initial pH, ^5^ Medium volume, ^6^ Rotation speed, ^7^ Fermentation. *p* ≤ 0.05 and *p* ≤ 0.01 were considered statistically significant difference and extremely significant difference, respectively.

**Table 3:** Test of significant effects of five factors on fermentation for TYR production from Rac56 using Data Processing System.

^1^ TYR											
T_1_	13.7101	15.9705	15.8391	15.244	13.4073	^2^ V	SS	df	S^2^	*F*	*p*
T_2_	19.664	16.223	15.9399	14.6122	13.4654	^3^ TE	2.1205	3	0.7068	21.3276	0.0001
T_3_	15.1039	14.2837	14.0013	16.6605	17.3406	^4^ pH	0.1871	3	0.0624	1.8822	0.1525
T_4_	13.319	15.3198	16.0167	15.2803	17.5837	^5^ MV	0.2343	3	0.0781	2.3563	0.0903
*x*_1_	1.1425	1.3309	1.3199	1.2703	1.1173	^6^ RS	0.1865	3	0.0622	1.8762	0.1535
*x*_2_	1.6387	1.3519	1.3283	1.2177	1.1221	^7^ FT	1.3532	3	0.4511	13.6102	0.0001
*x*_3_	1.2587	1.1903	1.1668	1.3884	1.4451	error	1.0605	32	0.0331		
*x*_4_	1.1099	1.2767	1.3347	1.2734	1.4653	*F*_0.05_	2.90				
R	0.5288	0.1616	0.1679	0.1707	0.3480	*F*_0.01_	4.46				

^1^ *p*-Tyrosol, ^2^ Source of Variation, ^3^ Temperature, ^4^ Initial pH, ^5^ Medium volume, ^6^ Rotation speed, ^7^ Fermentation. *p* ≤ 0.05 and *p* ≤ 0.01 were considered statistically significant difference and extremely significant difference, respectively.

**Table 4:** Results of an orthogonal-design experiment to determine the optimal fermentation conditions for SAL and TYR production from Rac56 using Data Processing System.

Codes	Factors					Content (mg/mL)	
	TE ^1^	pH ^2^	MV ^3^	RS ^4^	FT ^5^	SAL ^6^	TYR ^7^
1	20	5	25	100	3	1.6264 ± 0.1482	1.0312 ± 0.0071
2	20	6	50	150	7	1.9082 ± 0.0720	1.0128 ± 0.0069
3	20	7	100	200	11	1.2076 ± 0.1359	1.1833 ± 0.0524
4	20	8	150	250	15	1.6904 ± 0.0822	1.3428 ± 0.2233
5	25	5	50	200	15	1.9383 ± 0.0475	2.0018 ± 0.0009
6	25	6	25	250	11	2.0502 ± 0.0477	1.8792 ± 0.0860
7	25	7	150	100	7	2.3392 ± 0.1093	1.4064 ± 0.1196
8	25	8	100	150	3	1.7475 ± 0.0789	1.2673 ± 0.0111
9	30	5	100	250	7	1.4483 ± 0.1551	1.0020 ± 0.0014
10	30	6	150	200	3	1.3964 ± 0.0129	1.3012 ± 0.1997
11	30	7	25	150	15	1.2126 ± 0.1286	1.3021 ± 0.2278
12	30	8	50	100	11	1.1450 ± 0.1473	1.4293 ± 0.2049
13	35	5	150	150	11	1.3489 ± 0.2708	1.2885 ± 0.1504
14	35	6	100	100	15	1.2687 ± 0.1773	1.2145 ± 0.1107
15	35	7	50	250	3	1.3894 ± 0.0478	0.8694 ± 0.0961
16	35	8	25	200	7	1.6011 ± 0.0927	1.0672 ± 0.0888

Values are mean ± SD. ^1^ Temperature, ^2^ Initial pH, ^3^ Medium volume, ^4^ Rotation speed, ^5^ Fermentation time, ^6^ salidroside, ^7^ *p*-tyrosol.

**Table 5:** Five factors and their four levels in an L16(4^5^) orthogonal design.

Factor	Levels			
	1	2	3	4
Temperature (°C)	20	25	30	35
Initial pH	5	6	7	8
Medium volume (mL)	25	50	100	150
Rotation speed (rpm)	100	150	200	250
Fermentation time (day)	3	7	11	15

## Supplementary Materials

Supplementary materials can be accessed at: http://www.mdpi.com/1420-3049/21/4/502/s1.

## Abbreviations

The following abbreviations are used in this manuscript:

SAL: salidroside
YAL: *p*-tyrosol
DSE: dark septate endophytes
CD: Czapek-Dox medium
PD: potato dextrose medium
SD: sabouraud medium
LBP: lactose-beef extract-peptone medium
HPLC: high performance liquid chromography
UPLC/Q-TOF-MS: ultra-performance liquid chromatography/quadrupole time–off light mass spectrometry
^1^H-NMR: ^1^H nuclear magnetic resonance
