# Supplementary material for: Potential of the Endophytic Fungus Phialocephala fortinii Rac56 Found in Rhodiola Plants to Produce Salidroside and p-Tyrosol
Source: Molecules. 2016 Apr 16;21(4):502. doi: 10.3390/molecules21040502 (PMC6273359; doi:10.3390/molecules21040502)
Supplement: Supplementary file 1 [file molecules-21-00502-s001.pdf]

Supplementary Materials: Potential of the Endophytic Fungus *Phialocephala fortinii* Rac56 Found in *Rhodiola* Plants to Produce Salidroside and *p*-Tyrosol

Jinlong Cui, Tingting Guo, Jianbin Chao, Mengliang Wang and Junhong Wang

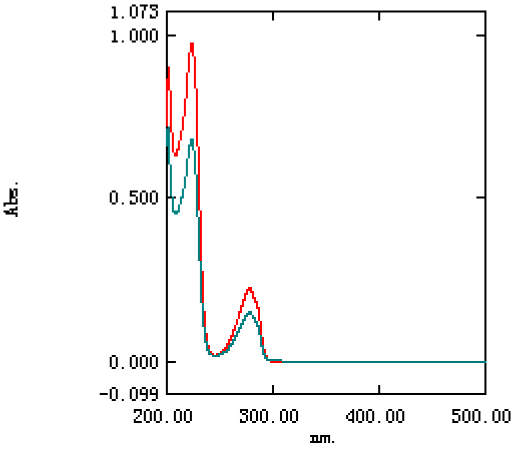

(**a**)

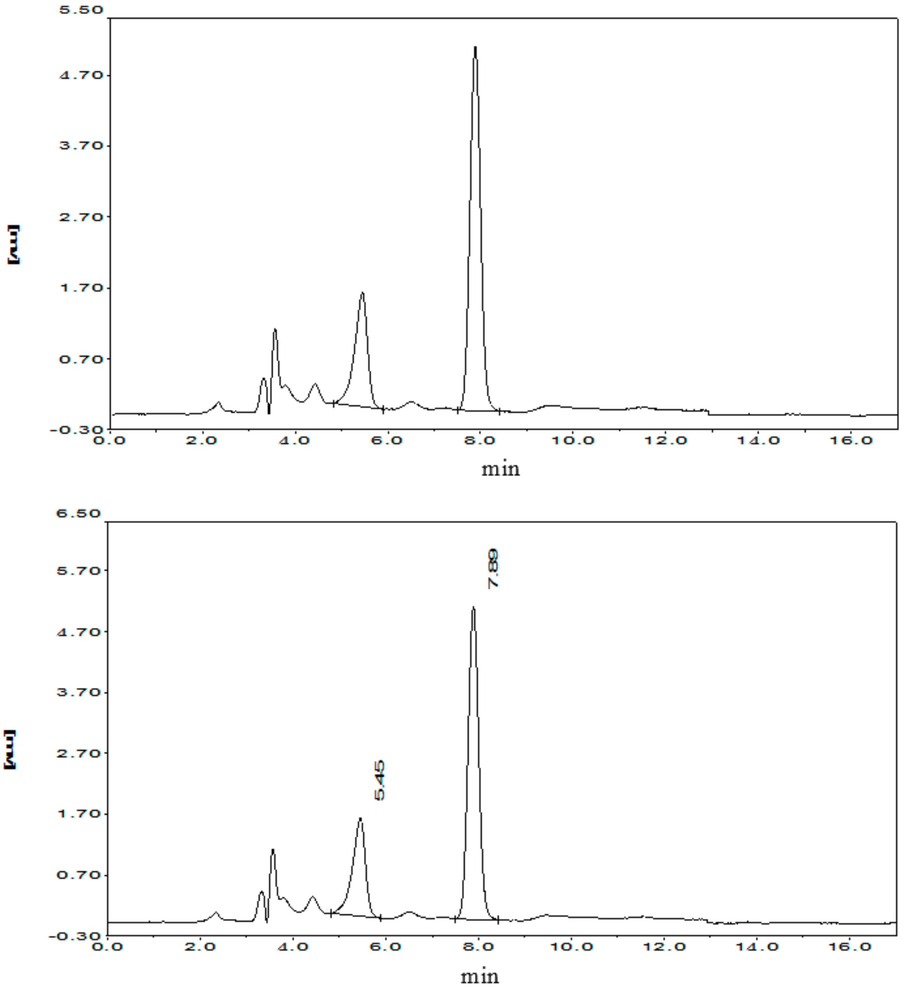

(**b**)

Figure S1. *Cont.*

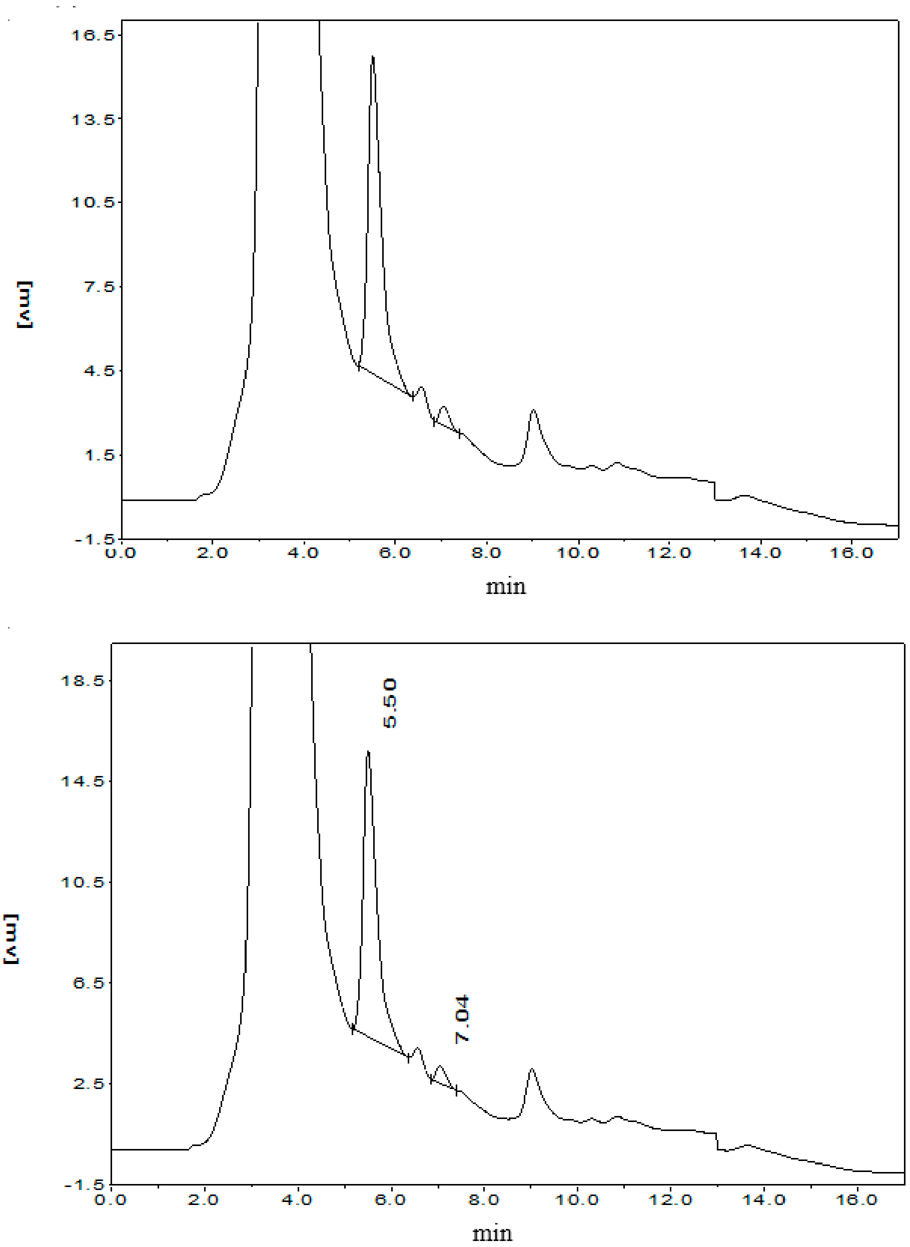

(c)

Figure S1. *Cont.*

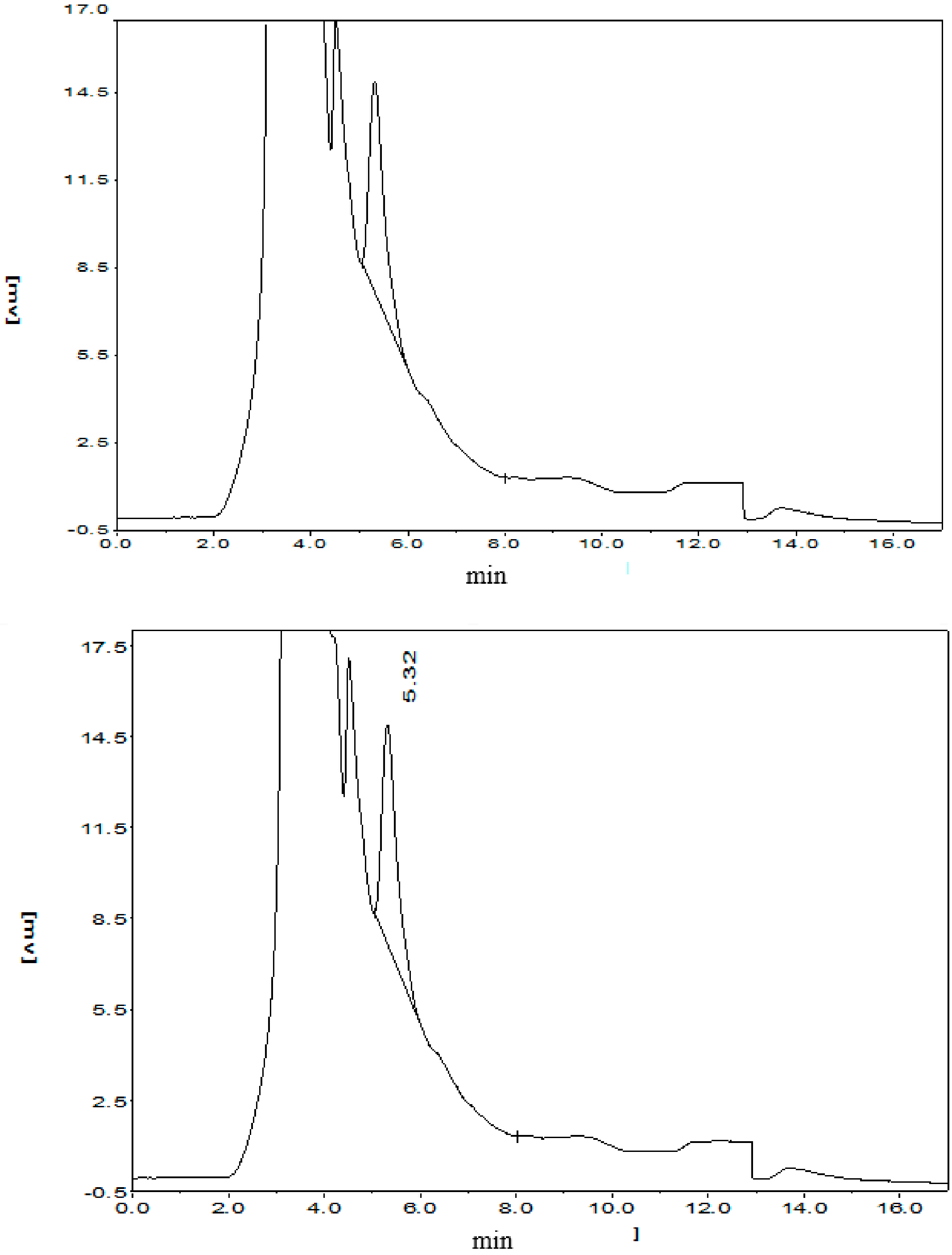

(**d**)

Figure S1. *Cont.*

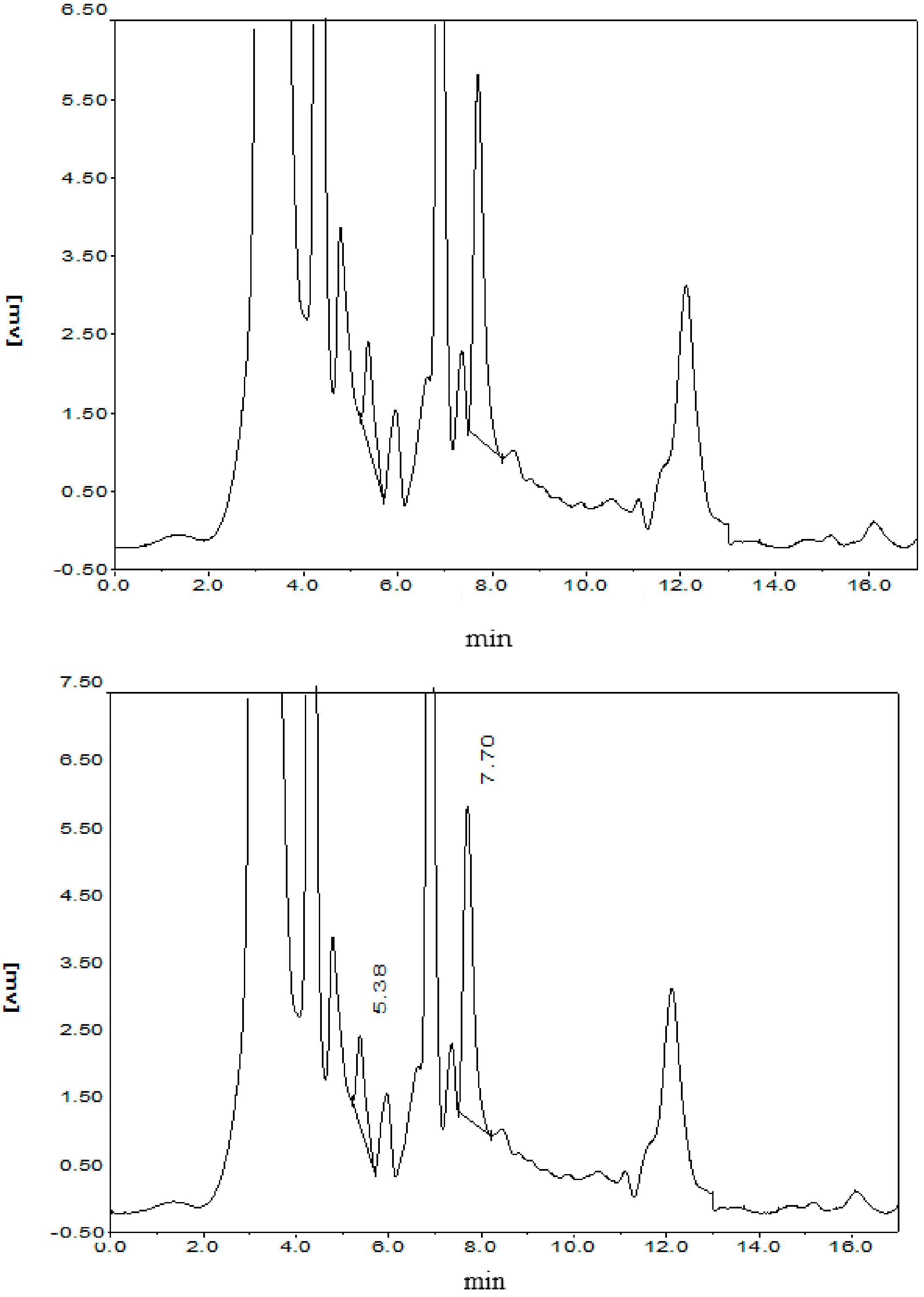

(e)

Figure S1. *Cont.*

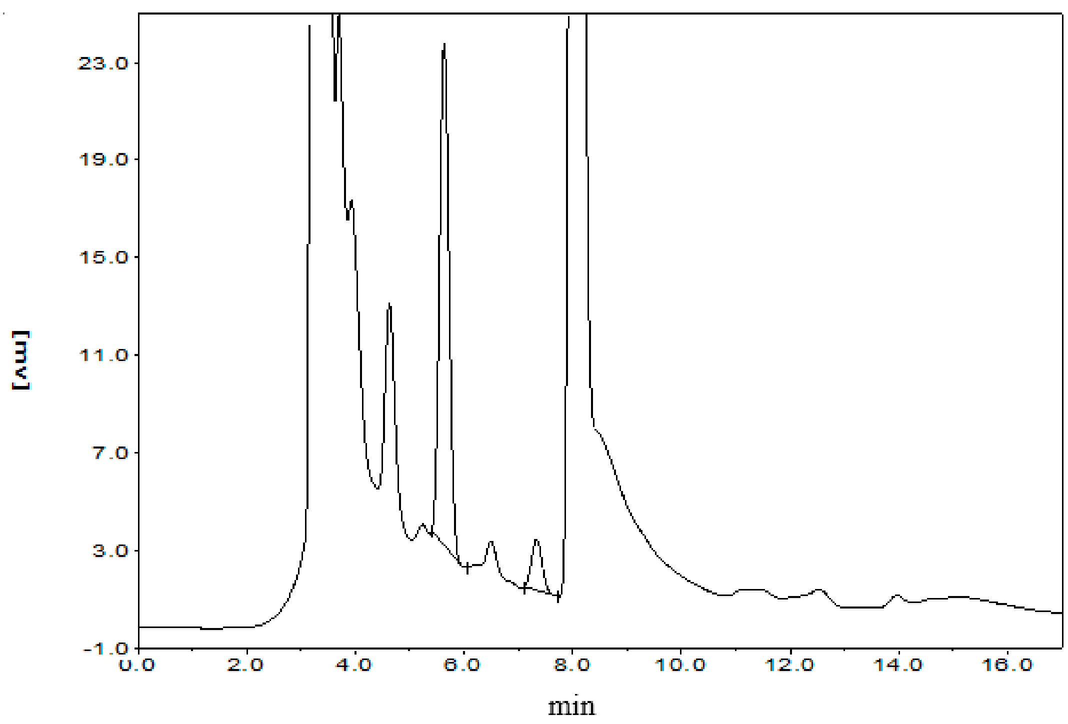

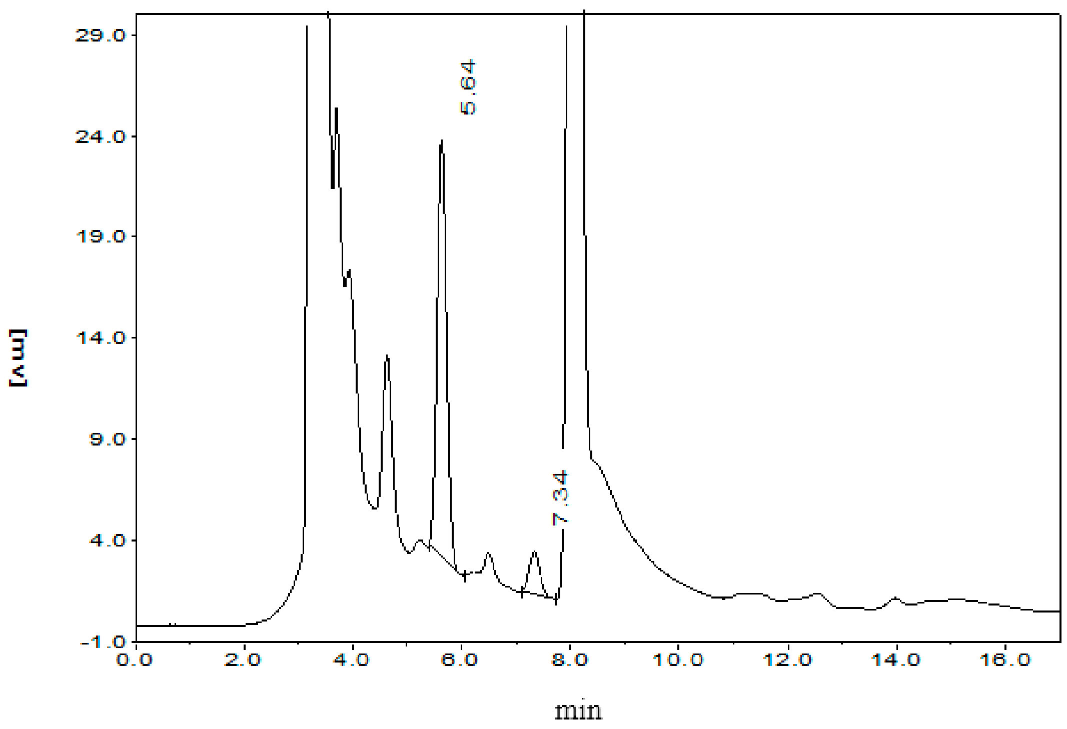

(**f**)

Figure A1. Ultraviolet absorption and high performance liquid chromatogram of substances. (**a**) Ultraviolet absorption. (**b**) Authentic salidroside (SAL) and p-tyrosol (TYR) and fungal SAL and TYR. (**c**–**f**) were the records of Rac12 (mycelial extract), Rct30 (mycelial extract), Rac56 (filtrate extract) and Rac63 (filtrate extract), respectively. The mobile phase consisted of methanol (Solvent A):water (Solvent B) = 30:70 at a flow rate of 1.0 mL/min at 30 °C was monitored using a UV-detector at 276 nm. Four fungal samples showed peaks of SAL and TYR with identical retention times as the authentic SAL and TYR.
